# A Cross-Sectional Survey of Fixed-Dose Combination Antihypertensive Medicine Prescribing in Twenty-Four Countries, Including Qualitative Insights

**DOI:** 10.5334/gh.1353

**Published:** 2024-09-12

**Authors:** Edel O’Hagan, Daniel McIntyre, Tu Nguyen, Kit Mun Tan, Peter Hanlon, Maha Siddiqui, Dzudie Anastase, Toon Wei Lim, Anezi Uzendu, Tan Van Nguyen, Wei Jin Wong, Hui Min Khor, Pramod Kumar, Timothy Usherwood, Clara K. Chow

**Affiliations:** 1 Westmead Applied Research Centre, The University of Sydney, Entrance K, Level 5, Westmead Hospital, Hawkesbury Road, Westmead, NSW, 2145, Australia; 2 The George Institute for Global Health, Level 18, International Towers 3, 300 Barangaroo Ave, Sydney, NSW, 2000, Australia; 3 Sydney School of Public Health, The University of Sydney, Edward Ford Building, A27 Fisher Rd Camperdown, NSW, 2050, Australia; 4 Division of Geriatric Medicine, Department of Medicine, Faculty of Medicine, Universiti Malaya, 50603 Kuala Lumpur, Malaysia; 5 School of Health and Wellbeing, University of Glasgow, Clarice Pears building, Byres Road, Glasgow, UK; 6 Clinical Research Education, Networking and Consultancy and Department of Medicine, Douala General Hospital, Douala, Cameroon; 7 National University Heart Centre, National University Hospital, 5 Lower Kent Ridge Road, 119074, Singapore; 8 Department of Internal Medicine, University of Texas Southwestern Medical Center, 5323 Harry Hines Blvd., Dallas, TX, 75390, United States; 9 University of Medicine & Pharmacy at Ho Chi Minh City, Ho Chi Min City, Vietnam; 10 School of Pharmacy, Monash University Malaysia, Jalan Lagoon Selatan, 47500 Bandar Sunway, Subang Jaya, Selangor, Malaysia; 11 All India Institute of Medical Sciences, Sri Aurobindo Marg, Ansari Nagar, Ansari Nagar East, New Delhi, Delhi 110029, India

**Keywords:** hypertension, antihypertensive medications, fixed dose combinations, global healthcare survey, polypills

## Abstract

**Background:**

Treatment inertia, non-adherence and non-persistence to medical treatment contribute to poor blood pressure (BP) control worldwide. Fixed dose combination (FDC) antihypertensive medicines simplify prescribing patterns and improve adherence. The aim of this study was to identify factors associated with prescribing FDC antihypertensive medicines and to understand if these factors differ among doctors worldwide.

**Methods:**

A cross-sectional survey was conducted online from June 2023 to January 2024 to recruit doctors. We collaborated with an international network of researchers and clinicians identified through institutional connections. A passive snowballing recruitment strategy was employed, where network members forwarded the survey link to their clinical colleagues. The survey instrument, developed through a literature review, interviews with academic and clinical researchers, and pilot testing, assessed participants perspectives on prescribing FDC antihypertensive medicines for hypertension. Participants rated their level of agreement (5-point Likert scale) with statements representing six barriers and four facilitators to FDC use.

**Findings:**

Data from 191 surveys were available for analysis. 25% (n = 47) of participants worked in high-income countries, 38% (n = 73) in upper-middle income, 25% (n = 48) in lower-middle income, 6% (n = 10) in low-income countries. Forty percent (n = 70) of participants were between 36–45 years of age; two thirds were male. Cost was reported as a barrier to prescribing FDC antihypertensive medicines [51% (n = 87) agreeing or strongly agreeing], followed by doctors’ confidence in BP measured in clinic [40%, (n = 70)], access [37%, (n = 67)], appointment duration [35%, (n = 61)], concerns about side-effects [(21%, n = 37)], and non-adherence [12%, (n = 21)]. Facilitators to FDC antihypertensive polypills prescribing were clinician facing, such as access to educational supports [79%, (n = 143)], more BP measurement data [67%, (n = 120)], a clinical nudge in health records [61%, (n = 109)] and patient-facing including improved patient health literacy [49%, (n = 88)]. The levels of agreement and strong agreement across all barriers and facilitators were similar for participants working in higher or lower income countries. Across all countries, participants rated FDC antihypertensive medications highly valuable for managing patients with non-adherence, (82% reported high or very high value), for patients with high pill burden (80%).

**Interpretation:**

Cost and access were the most common barriers to prescribing FDCs across high- and low-income countries. While greater educational support for clinicians was perceived as the leading potential facilitator of FDC use, this seems unlikely to be effective without addressing access.

## Introduction

Hypertension is the leading contributor to premature death globally (1), yet poorly controlled worldwide. In 2019, half of those with hypertension were aware of their condition, yet less than a quarter had their blood pressure (BP) controlled (2), with rates similar to a decade earlier (3).

Approximately 75% of hypertensive patients live in low- and middle-income countries where rates of awareness and control are particularly low (4).

Treatment inertia, the lack of up-titration of medications despite treatment targets not being reached is a major impediment to hypertension control (5,6,7) International studies report treatment inertia in as many as 60% of clinic visits where patients presented with uncontrolled BP in the United States (7), 58% of similar visits in Spain (8), and 65% of similar visits in the Dominican Republic (9). Treatment inertia is exacerbated in patients who require multiple medications to achieve targets, and when there are concerns regarding adverse events (10). Sub-optimal adherence and non-persistence also contribute to poor blood pressure control (11,12). A large meta-analysis of prospective epidemiologic studies linked up to 9% of cardiovascular disease events to poor adherence (13). Complex dosing regimens and polypharmacy are associated with non-adherence (14).

Fixed-dose combination (FDC) pills simplify treatment regimens (15) and have been shown in randomized clinical trials (RCTs) to improve adherence to BP-lowering therapy (16). There is also increasing research demonstrating safety and efficacy of triple and quadruple combination BP-lowering FDC medicines (17). A recent exploratory analysis of the QUARTET RCT demonstrated that there was less treatment inertia among patients using the quad pill (FDC pill containing four ultra-low doses of BP-lowering drugs) compared to patients on monotherapy (18). Though this was different from an analysis of TRIUMPH, an unblinded RCT of low-dose triple-combination FDC, that observed higher treatment inertia rates among the intervention group (15). This discrepancy suggests that while FDC antihypertensive polypills offer a convenient and scalable management strategy, their impact on treatment inertia may vary across countries due to factors beyond medication characteristics.

The growing body of evidence on poor BP control, the need for combination BP lowering to achieve BP control, and the efficacy and safety of FDCs have led to recent updates to European guidelines on BP management, and the US Society of Hypertension recommend initial treatment with dual combination medicine (19,20). Yet the persisting lack of adoption of FDCs worldwide (3,21,22,23) calls for further research into why FDCs are not utilized. The aim of this study was to identify factors associated with prescribing FDC antihypertensive medicines to patients with hypertension, and to understand if these factors differ among doctors working in low-, middle- or high-income countries.

## Methods

### Study design

This was a cross-sectional online global survey, whereby the countries in which the participants worked were categorized based on their World Bank classification at study initiation (24). The University of Sydney Human Research Ethics Committee (2023/362) approved the study. The trial protocol was registered on the Open Science Framework prior to recruitment (25). This study was reported following the STROBE reporting guideline for cross-sectional studies (Supplementary Material 1). A participant information and informed consent document was available for download; participants were required to acknowledge that they read and accepted the consent before commencing the online survey.

### Setting and participants

The survey was created in Qualtrics™ (26) and distributed online using convenience non-probability sampling. We collaborated with an international network of researchers, research clinicians and clinicians identified through institutional connections. We employed a passive snowballing recruitment strategy, whereby we encouraged members of this network to share the survey with their clinical colleagues, but we did not directly request the clinicians’ contact details. The survey was open for six months between June–December 2023 and accessed via direct email. Recruitment efforts were supplemented with fortnightly updates to international collaborators to encourage continued promotion of the survey. Eligible participants were medical doctors, who as part of their usual medical practice prescribed antihypertensive medications to patients with high blood pressure. Participants from any country were eligible for inclusion.

### Survey instrument

The survey instrument was developed in an iterative process with members of the research team, including academic and clinical researchers. We conducted a literature review to inform the possible barriers and facilitators to polypill prescribing internationally (27,28,29). We then developed an initial draft of the survey, using established design principles (30,31). Subsequently, we conducted an interview with prescribing doctors and members of our research team (family physician [TU], cardiologist [CK], and gerontologist [TN]), to further refine items to assure content validity and wording clarity. The survey was pilot tested on a group of participants who were representative of the target population (n = 16). The feedback received was analyzed and the survey was refined. Due to the known challenges of recruiting doctors to complete surveys (32,33,34), the survey was optimized for brevity and ease of completion. The final version was pilot tested again with clinicians prior to commencing recruitment. The full survey is available in Supplementary Material 2. We commenced recruitment using an English language version of the survey and translated the survey into the six UN languages (English, French, Arabic, Russian, Chinese, Spanish) to enable international recruitment. The survey was translated using backtranslation, whereby the original survey was translated to one of the six languages by a native speaker and the translation back translated into English (35).

### Variables

In this study, fixed-dose combination referred to single-pill combination medicines, where two or more antihypertensive medicines were combined. Participants rated their agreement (5-point scale, with a range from 0 indicating strong disagreement to 5 indicating strong agreement) with six statements to represent barriers and four statements to represent facilitators to FDC antihypertensive medicine prescribing for patients with hypertension. Demographic characteristics, geographic location, clinical experience, and current prescribing practices, including frequency of prescribing FDC medicines for the last 10 patients treated with hypertension were self-reported. Additionally, participants rated the value of a FDC antihypertensive medicine in various clinical scenarios (5-point scale: 1-very low value, 5 = high value) and importance of different factors influencing their decision to initiate FDC antihypertensive therapy (5-point scale: 1 = not at all important, 5 = extremely important). Finally, an open-ended question invited participants to share additional comments on barriers and facilitators to FDC antihypertensive medicine use for the control of hypertension.

### Data analysis

Continuous variables are presented through centrality measures (mean, median), and dispersion (SD and IQR) according to the distribution, and categorical variables through frequencies and percentages. Data from physicians who work in high-income countries and upper-middle-income countries were combined, lower-middle-income countries and low-income countries were combined to create a *higher income* and *lower income* groups (36). Having two broader groups (high and low) enhanced statistical power while capturing significant variations in socioeconomic contexts.

The median and IQR score for agreement with each barrier and facilitator is presented. We calculated the standardized mean difference in scores between participants from higher-income countries or lower-income countries. These results are presented along with the corresponding confidence intervals. We examined the relationship between FDC prescribing frequency and self-reported barriers and facilitators to FDC use. We examined whether this relationship varied by whether the participant worked in a higher or lower income country using interaction analysis. The value of FDC antihypertensive medication and considerations for initiating such therapy in hypothetical clinical scenarios were described and presented as proportions. Analysis was performed in the R environment for statistical computing, version 4.3.2 (37).

One researcher (EO) performed a thematic analysis on the responses to the open-ended question to understand participants’ perspective and interpreted the comments alongside the demographic, clinical and prescribing practices data. The interpretation was reviewed by another author (DM).

### Role of the funding source

This research did not receive any external funding or financial support from any organization or institution.

## Results

Between June 2023 and January 2024, we recruited registered doctors who prescribed BP-lowering medicines. The participants trained in 32 different countries and work in 24 different countries. Of the countries where the participants work, 21 of the countries were higher-income, and 11 were lower-income countries.

**Figure 1:**
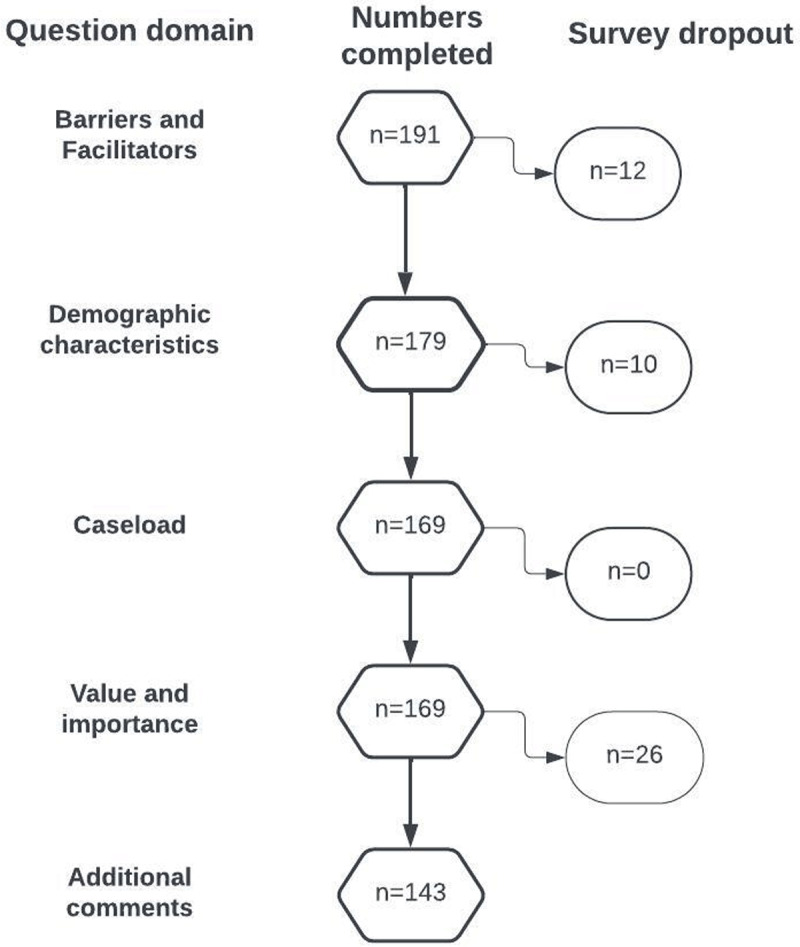
Number of participants who completed each block of survey questions.

**Figure 2:**
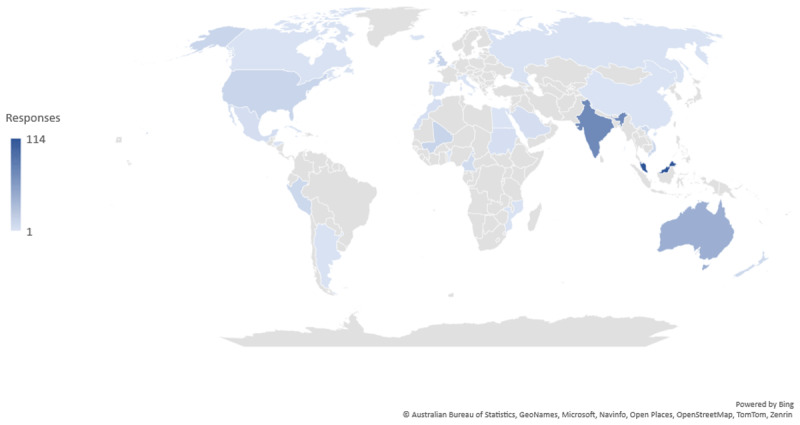
Map of countries represented. Number of participants per country: Argentina (n = 1), Australia (n = 21), Bahamas (n = 1), Bahrain (n = 2), Belgium (n = 1), Cote d’Ivoire (n = 2), Cameroon (n = 5), Honduras (n = 1), India (n = 34), Jamaica (n = 1), Malaysia (n = 64), Mali (n = 8), Mexico (n = 3), Mozambique (n = 1), New Zealand (n = 6), Peru (n = 5), Saint Kitts (n = 1), Saudi Arabia (n = 4), Singapore (n = 2), Spain (n = 1), Sudan (n = 2), United Kingdom (n = 3), United States of America (n = 5), Viet Nam (n = 5).

We had complete data from 191 surveys in total and missing data across 48 surveys. Figure 1 shows the numbers of participants analyzed for each block of survey questions: 25% (n = 47) of participants worked in high income countries, 38% (n = 73) worked in upper-middle income countries, 25% (n = 48) worked in lower-middle income countries, 6% (n = 10) worked in low-income countries, and 6% (n = 12) did not complete that question in the survey. Figure 2 shows a world map with the countries that were represented in the survey; a full list is available in Supplementary Material 3, Table 1.

One third (n = 55) of participants were between 26–35 years, and 40% (n = 70) were between 36–45 years of age; 62% (n = 111) were male. Participants had 13 years (IQR 8, 20) of experience. The majority of participants were geriatricians, 29% (n = 52), family practitioners, 25% (n = 45), or cardiologists, 22% (n = 40). Participants estimated that 28% (IQR 16, 39) of the patients with known hypertension that they reviewed in their last working week were on an initial monotherapy antihypertensive regimen, whereas 22% (IQR 8, 30) were on a single pill combination of two BP medicines, 8% (IQR 0, 11) were on a single pill combination of three or more medicines, 26% (IQR 17, 35) were on two blood pressure lowering medicines separately and 16% (IQR 5, 23) on three or more BP-lowering medicines separately. For those on initial monotherapy, 32% (IQR 21, 38) were taking an ACE inhibitor or ARB and 30% (IQR 19, 42) a dihydropyridine calcium channel blocker. Full details of the demographic characteristics, clinical experience and current prescribing practices are available in Table 1.

**Table 1:** Participant characteristics, clinical experience, and prescribing patterns.


	OVERALL COHORT (n = 179)	HIGH INCOME COUNTRIES (GROSS NATIONAL INCOME (GNI) PER CAPITA IN US DOLLARS $13,846 OR MORE) (n = 47)	UPPER MIDDLE-INCOME COUNTRIES (GNI $4,466USD TO $13,845) (n = 73)	LOWER MIDDLE-INCOME COUNTRIES (GNI $1,136USD TO $4,465) (n = 49)	LOW-INCOME COUNTRIES (GNI $1.135USD OR LESS) (n = 10)

**Age, (n (%))**					

25 years or less	1 (1%)	1 (2%)	0	0	0

26–35years	55 (31%)	8 (17%)	19 (26%)	27 (56%)	1 (9%)

36–45 years	71 (40%)	14 (30%)	39 (53%)	14 (29%)	4 (36%)

46–55 years	30 (17%)	10 (21%)	11 (15%)	4 (8%)	5 (45%)

56–65 years	14 (8%)	10 (21%)	3 (4%)	1 (2%)	0

66+ years	8 (5%)	0	0	0	0

**Gender, (n (%))**					

Male	111 (62%)	29 (62%)	40 (55%)	34 (69%)	8 (80%)

Female	68 (38%)	17 (36%)	33 (45%)	15 (31%)	2 (20%)

Non-binary/third gender	0	1 (2%)	0	0	0

**Years practicing, median (IQR)**	13 (8, 20)	18 (10, 30)	14 (10, 19)	9 (5, 15)	20 (16, 22)

**Specialty, (n (%))**					

Cardiologist	40 (22%)	13 (28%)	8 (11%)	10 (20%)	9 (90%)

Endocrinologist	3 (2%)	0	3 (4%)	0	0

Family Practitioner	45 (25%)	27 (57%)	11 (15%)	7 (15%)	0

Geriatrician	52 (29%)	4 (9%)	21 (29%)	27 (56%)	0

Nephrologist	4(2%)	1 (2%)	3 (4%)	0	0

Anaesthetist	1 (1%)	0	1 (2%)	0	0

Emergency	1 (1%)	0	1 (2%)	0	0

General Medicine	10 (6%)	1 (2%)	7 (15%)	1 (2%)	1 (9%)

Internal Medicine	12 (7%)	0	9 (19%)	3 (6%)	

Neurology	4 (2%)	0	4 (8%)	0	

Neurosurgery	1 (1%)	0	1 (2%)	0	

Rehab	3 (2%)	1 (2%)	2 (4%)	0	

Rheumatology	3 (2%)	0	2 (4%)	1 (2%)	

**Patients per week, (n (%))**					

0–20	78 (44%)	26 (57%)	39 (53%)	8 (16%)	5 (50%)

21–40	45 (25%)	11 (24%)	19 (26%)	13 (27%)	2 (20%)

41–60	24 (13%)	5 (11%)	9 (12%)	9 (19%)	1 (10%)

61–80	13 (7%)	2 (4%)	2 (3%)	8 (17%)	1 (10%)

81–100	7 (4%)	1 (2%)	3 (4%)	2 (4%)	1 (10%)

100+	11 (6%)	1 (2%)	1 (1%)	9 (19%)	0

**Considering your last working week, what percentage of the patients you saw were on the following antihypertensive regimens: [n; mean (IQR)]**					

Initial monotherapy	160; 28 (16, 39)	44; 29 (19, 42)	68; 29 (16, 43)	41; 27 (15, 34)	7; 24 (10, 35)

Single pill combination of two medicines	160; 22 (8, 30)	44; 20 (10, 27)	68; 15 (3, 21)	41; 31 (19, 39)	7; 46 (33, 52)

Single pill combination of three or more medicines	160; 8 (0, 11)	44; 10 (0, 18)	68; 6 (0, 8)	41; 9 (2, 10)	7; 11 (3, 16)

Two blood pressure lowering medicines separately	160; 26 (17, 35)	44; 26 (15, 40)	68; 30 (21, 37)	41; 22 (15, 29)	7; 16 (9, 23)

Three or more blood pressure lowering medicines separately	160; 16 (5, 23)	44; 15 (6, 21)	68; 20 (10, 30)	41; 12 (4, 21)	7; 3 (0, 5)

**Thinking about the patients you saw last week on single drug monotherapy; what percentage are on each on the following: [n; mean (IQR)]**					

ACE inhibitor or ARB (Ramipril or telmisartan)	160; 33 (21, 38)	44; 38 (23, 55)	68; 32 (22, 33)	41; 28 (20, 36)	7; 32 (7, 22)

Dihydropyridine calcium channel blocker (amlodipine)	160; 31 (19, 42)	44; 28 (10, 29)	68; 29 (22, 41)	41; 36 (23, 45)	7; 29 (25, 60)

Thiazide-like diuretic (e.g. hydrochlorothiazide)	160; 10 (1, 15)	44; 7 (1, 17)	68; 13 (1, 10)	41; 9 (7, 15)	7; 7 (1, 8)

Loop diuretic (e.g. furosemide)	160; 6 (0, 10)	44; 5 (0, 9)	68; 6 (0, 12)	41; 6 (1, 8)	7; 11 (0, 3)

Beta blocker (e.g. atenolol. metoprolol)	160; 14 (7, 20)	44; 13 (5, 16)	68; 14 (8, 23)	41; 14 (7, 17)	7; 14 (8, 23)

Other blood pressure lowering medicine	160; 7 (1, 10)	44; 9 (1, 10)	68; 6 (0, 10)	41;7 (3, 10)	7; 7 (2, 8)


Cost was the most commonly selected barrier to prescribing FDC (n = 87, 51%, agreed or strongly agreed) followed by confidence in clinic BP measurement (n = 70, 40% agreed or strongly agreed), access to FDC medicines (n = 67, 37% agreed or strongly agreed), appointment duration (n = 61, 35% agreed or strongly agreed), patient concerns about side-effects (n = 37, 21% agreed or strongly agreed) and concerns about patient non-adherence (n = 21, 12% agreed or strongly agreed). The levels of agreement and strong agreement across all barriers were the same for participants working in higher or lower income countries, with no difference between groups. These results are presented in Table 2 and Supplementary Material 4, Figure 1.

**Table 2:** Barriers and facilitators to prescribing for doctors in high- and upper-middle income or lower-middle and low-income countries.


STATEMENTS ABOUT BARRIERS AND FACILITATORS TO PRESCRIBING FIXED-DOSE COMBINATION ANTIHYPERTENSIVE MEDICATIONS FOR THE CONTROL OF HYPERTENSION	ALL (INCLUDING DATA WHERE COUNTRY OF WORK WAS NOT AVAILABLE) n = 179	HIGHER INCOME n = 120	LOWER INCOME n = 59	STANDARDIZED MEAN DIFFERENCE (BETWEEN HIGHER AND LOWER INCOME COUNTRIES)	95% CONFIDENCE INTERVAL

**Appointment Time** **You have insufficient time during consultations to explain medication changes to the patient**.					

*Strongly disagree*	35 (20%)	23 (19%)	12 (20%)	0.01	–0.30 to 0.32

*Disagree*	61 (34%)	39 (32%)	22 (37%)		

*Neither disagree nor agree*	21 (12%)	18 (15%)	3 (5%)		

*Agree*	55 (31%)	36 (30%)	19 (32%)		

*Strongly agree*	7 (4%)	4 (3%)	3 (5%)		

**Access** **Your patients do not have access to fixed-dose combination antihypertensive medications where you work**.					

*Strongly disagree*	57 (32%)	34 (28%)	23 (39%)	0.08	–0.23 to 0.39

*Disagree*	40 (22%)	28 (23%0	12 (20%)		

*Neither disagree nor agree*	15 (8%)	13 (11%)	2 (3%)		

*Agree*	38 (21%)	28 (23%)	10 (17%)		

*Strongly agree*	29 (16%)	17 (14%)	12 (20%)		

**Confidence in clinic BP measurement** **You do not trust blood pressure measurements taken in the clinic setting**.					

*Strongly disagree*	34 (19%)	22 (18%)	12 (20%)	0.14	–0.17 to 0.45

*Disagree*	47 (26%)	29 (24%)	18 (31%)		

*Neither disagree nor agree*	28 (16%)	21 (18%)	7 (12%)		

*Agree*	62 (35%)	41 (34%)	21 (36%)		

*Strongly agree*	8 (5%)	7 (6%)	1 (2%)		

**Cost to the medical practice** **Fixed-dose combination antihypertensive medications are too expensive for your medical practice to support**.					

*Strongly disagree*	23 (13%)	18 (16%)	5 (9%)	0.07	–0.24 to 0.39

*Disagree*	42 (25%)	23 (20%)	19 (33%)		

*Neither disagree nor agree*	19 (11%)	13 (11%)	6 (11%)		

*Agree*	60 (35%)	40 (35%)	20 (35%)		

*Strongly agree*	27 (16%)	23 (18%)	7 (12%)		

**Side effects** **Your patients are concerned about experiencing more adverse events (for example, dizziness, headache) when taking fixed-dose combination antihypertensive medications compared to adding medicines sequentially**.					

*Strongly disagree*	30 (17%)	19 (16%)	11 (19%)	–0.32	–0.64 to –0.01

*Disagree*	68 (38%)	52 (43%)	16 (27%)		

*Neither disagree nor agree*	44 (25%)	32 (27%)	12 (20%)		

*Agree*	31 (17%)	15 (12%)	16 (27%)		

*Strongly agree*	6 (4%)	2 (2%)	4 (7%)		

**Adherence** **You are concerned your patients will not adhere to the fixed-dose combination medication regimen**.					

*Strongly disagree*	71 (40%)	47 (39%)	24 (41%)	–0.06	–0.37 to 0.25

*Disagree*	72 (40%)	48 (40%)	24 (41%)		

*Neither disagree nor agree*	15 (9%)	12 (10%)	3 (5%)		

*Agree*	15 (9%)	12 (10%)	3 (5%)		

*Strongly agree*	6 (3%)	1 (1%)	5 (9%)		

**Clinician nudge** **A clinician nudge that provides a prompt in the electronic health record during the patient visit to prescribe fixed-dose combination antihypertensive medications would support the prescription of those medications**.					

*Strongly disagree*	8 (5%)	8 (7%)	0	–0.13	–.44 to 0.19

*Disagree*	15 (8%)	11 (9%)	4 (7%)		

*Neither disagree nor agree*	47 (26%)	27 (22%)	20 (34%)		

*Agree*	80 (45%)	54 (45%)	26 (44%)		

*Strongly agree*	29 (16%)	20 (17%)	9 (15%)		

**Education** **Providing education and feedback on prescribing patterns compared with peers focusing on fixed-dose combination antihypertensive medications would support the prescription of those medications**.					

*Strongly disagree*	9 (5%)	6 (5%)	3 (5%)	0.01	–0.30 to 0.33

*Disagree*	10 (6%)	6 (5%)	4 (7%)		

*Neither disagree nor agree*	17 (10%)	11 (9%)	6 (10%)		

*Agree*	59 (33%)	42 (35%)	17 (29%)		

*Strongly agree*	84 (46%)	55 (46%)	29 (49%)		

**Additional BP measures data** **Having access to data from remote monitoring devices such as smartwatches would support the prescription of fixed-dose combination antihypertensive medications**.					

*Strongly disagree*	9 (5%)	6 (5%)	3 (5%)	0.23	–0.08 to 0.55

*Disagree*	24 (13%)	13 (11%)	11 (19%)		

*Neither disagree nor agree*	16 (15%)	18 (15%)	8 (14%)		

*Agree*	79 (44%)	51 (42%)	28 (47%)		

*Strongly agree*	41 (23%)	32 (27%)	9 (15%)		

**Health literacy** **Patient information such as leaflets and flyers in clinics focusing on fixed-dose combination antihypertensive medications would support the prescription of those medications**.					

*Strongly disagree*	18 (10%)	12 (10%)	6 (10%)	0.03	–0.29 to 0.34

*Disagree*	22 (12%)	15 (12%)	7 (12%)		

*Neither disagree nor agree*	51 (28%)	34 (28%)	17 (29%)		

*Agree*	66 (37%)	43 (36%)	23 (39%)		

*Strongly agree*	22 (12%)	16 (13%)	6 (10%)		


Access to educational supports such as feedback on prescribing patterns compared with peers was the most commonly selected facilitator to prescribing FDC (n = 143, 79% agreed or strongly agreed), followed by more BP measurement data (n = 120, 67% agreed or strongly agreed), a clinical nudge or prompt in a health record (n = 109, 61% agreed or strongly agreed), and improved patient health literacy (n = 88, 49% agreed or strongly agreed). The levels of agreement and strong agreement across all facilitators were the same for participants working in higher- or lower-income countries, with no difference between groups. These results are presented in Table 2 and Supplementary Material 4, Figure 1.

Both reported access and reported cost as barriers to prescribing FDCs were significantly associated with a lower prescription of FDC [access: β 0.58 (95% CI –0.86 to –0.29); cost β –0.81 (95% CI –1.14 to –0.50)]. Other investigated barriers or facilitators were not associated with prescription of FDC. There were no significant interactions with country income level of clinician (Table 3).

**Table 3:** Relationship between frequency of prescribing FDC antihypertensive medicines and barriers and facilitators.


BARRIER OR FACILITATOR	β	95% CONFIDENCE INTERVAL	P-VALUE	P-VALUE (95% CI) FOR INTERACTION WITH COUNTRY INCOME LEVEL

Appointment time	–0.06	–0.43 to 0.31	0.74	

Access	–0.58	–0.86 to –0.29	<0.001	0.22 (–0.28 to 0.71)

Confidence in blood pressure (BP) measurement	–0.35	–0.70 to 0.01	0.06	

Cost to medical practice	–0.81	–1.14 to –0.50	<0.001	0.02 (–0.57 to 0.61)

Side effects	–0.32	–0.74 to 0.10	0.13	

Adherence	0.00	–0.43 to 0.43	0.99	

Clinician nudge	0.22	–0.22 to 0.66	0.33	

Education	–0.10	–0.50 to 0.30	0.62	

Additional BP measures data	–0.17	–0.56 to 0.23	0.40	

Health literacy	0.11	–0.29 to 0.50	0.59	


Most participants were from Australia (n = 22), India (n = 34), or Malaysia (n = 64). We described the response of participants from these three countries to each barrier and facilitator and noted differences between Australia and Malaysia. In Australia 5% (n = 1) agreed or strongly agreed that access is a barrier to FDC antihypertensive medicines in comparison to 52% (n = 33) in Malaysia, similarly in relation to cost in Australia 14% (n = 3) agreed or strongly agreed that cost was a barrier whereas in Malaysia this was 70% (n = 45). These results are available in Supplementary Material 5.

**Figure 3:**
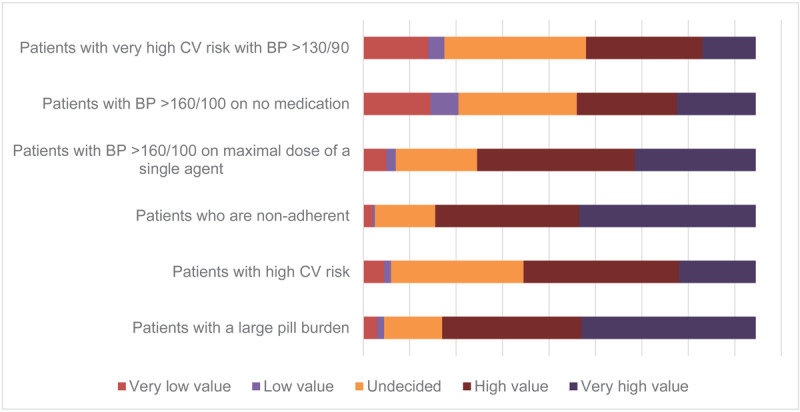
Value placed on fixed-dose combination antihypertensive medication in clinical scenarios.

Across all countries, participants rated FDC antihypertensive medications highly valuable for managing patients with non-adherence (82% reported high or very high value), for patients with high pill burden (80%). The proportion reporting highly valuable trended down across the categories of—for patients with uncontrolled hypertension (BP > 160/100 mmHg on maximal single-agent therapy) (71%), and for patients with very high cardiovascular risk with BP > 130/90 mmHg) (60%). Details are presented in Figure 3 and Supplementary Material 5, Table 2.

**Figure 4:**
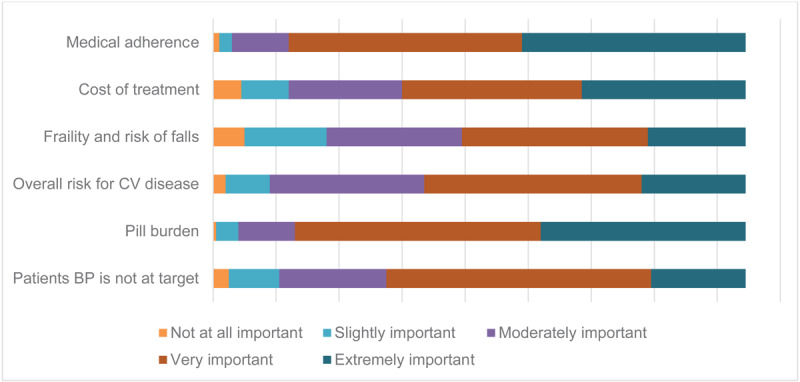
Factors important in the decision to initiate fixed-dose combination medicines.

With respect to initiating treatment with FDC antihypertensive therapy: Medical adherence (i.e., adherence to medications and medical advice such as lifestyle changes) held greatest importance (86%), followed by pill burden (85%), uncontrolled BP (67%), treatment cost (64%), overall CVD risk (60%), and frailty and risk of falls (53%). Details are presented in Figure 4 and Supplementary Material 5, Table 2.

There were open-ended question responses from 143 participants to the question, ‘What additional comments do you have regarding barriers and facilitators to the use of fixed-dose combination antihypertensive medications for the control of hypertension?’ The responses were distilled and organized into themes with quotes abstracted from the data. The quotes are presented with the participants country of practice, age range, and clinical speciality.

### Theme 1: Resource dependent

This theme incorporated the subthemes of cost and availability. The expense of antihypertensive medications was cited as a prohibitive barrier. This is interpreted from the quotes below.

Fixed-dose combinations are more expensive (StudyID 18, upper-middle-income country (Malaysia), 36–45 years old, Neurology)

These fixed-dose combinations are very expensive for the patient (StudyID 31, Lower-middle-income country (Cameroon), 26–35 years old, Family Practitioner)

Availability was also identified as a barrier. This is interpreted from the quote below.

There is need for widely available generic fixed dose combinations (StudyID 148, Upper-middle-income country (Jamaica), 46–55 years old, Internal Medicine)

Commonly cost and availability were reported together as barriers.

Main barrier in my working place would be cost and availability of FDC…. (StudyID 128, Upper-middle-income country (Malaysia), 26–35 years old, General Medicine)

Financial burden is one of the most important factors in prescription of medications in government settings. Patients wants to take fixed drug combinations, but they want medications free from hospital pharmacy where limited single drugs are available, limiting intake of field drug combinations (StudyID 116, Lower-middle-income country (India), 26–35 years old, Geriatrician)

### Theme 2: Challenges of a fixed dose

This theme incorporated the subthemes of concerns regarding dosage control and concerns regarding patient perceptions of fixed doses. A challenge with prescribing fixed-dose combinations is that doctors lose control over the dosage of each medicine. This theme was interpreted from the quotes below.

Prescribers are often concerned about side effects as there are not as many steps up or down in terms of dosages (StudyID 1, High-income country (St. Kitts and Nevis), 45–55 years old, Cardiologist)

Barrier to fixed-drug combo is when GP wants to add a second agent but the first anti-hypertensive and preferred second agent are not available in fixed-dose combo (StudyID 3, High-income country (Australia), 36–45 years old, Family Practitioner)

Difficulties with dose adjustment with fixed-dose antihypertensive medications (StudyID 148 Upper-middle-income country (Malaysia), 46–55 years old, Family Practitioner)

A participant reported on the challenge of managing patient expectations when prescribing medicines as a fixed dose.

Barrier would be patient perceptions on giving both medications without been able to adjust the dose (StudyID 136, Upper-middle-income country (Malaysia), 26–35 years old, General Medicine)

### Theme 3: Insufficient information

This theme incorporated the subthemes of concerns regarding side effects and insufficient education on fixed doses. Participants reported on fear of side effects, and difficulty determining which component is causing them. This theme was interpreted form the quotes below.

my concern is with side effects, a combination gives twice the opportunity to have side effects (StudyID 121, High-income country (New Zealand), 56–65 years old, Family Practitioner)

Other participants reported that they hadn’t sufficient guidance on when and how to use FDC medicines.

patient profile i.e. elderly, try not to use FDC (StudyID 20, Upper-middle-income country (Malaysia), 36–45 years old, Family Practitioner)

Information about misconception of long-term side effects on organ damage is lacking among our patients (StudyID 19, Upper-middle-income country (Malaysia), 36–45 years old, Family Practitioner)

One specifically recommended the following:

Physician education and product brochures (StudyID 10, Upper-middle-income country (Malaysia), 46–55 years old, Cardiologist)

## Discussion

In this analysis, we found that a high proportion of participants agreed or strongly agreed with statements related to cost, trust in clinic BP measurement, access, and appointment duration as barriers to prescribing FDC antihypertensive medicines, in descending priority. Participant responses showed a range of perspectives with considerable numbers choosing the ‘disagree’ and ‘strongly disagree’ categories in their response. Providing education and feedback on prescribing patterns compared with peers and access to data from remote monitoring devices could support the prescription of FDC antihypertensive medicines. These findings were consistent across high, middle and low-income country regions. Participants who prescribed FDC antihypertensive polypills more often were less inclined to report cost and access as barriers. FDC anti-hypertensive medicines were reported as valuable for patients with a large pill burden or for whom adherence is a problem, similarly medical adherence and pill burden were the most important factors to consider when thinking about initiating with FDC antihypertensive medicines. The qualitative findings mirror the quantitative results.

Cost emerged as the most consistently reported barrier to prescribing FDC antihypertensive medications, with this finding observed across participants from both higher and lower-income countries. This contrasts with prior analysis of availability and affordability of BP medicines across 21 high-, middle- and lower- income countries that found lower access to BP medicines in low-income settings, except for India (38). An analysis of the PURE study found substantial disparities in access to essential BP medicines (β-blockers, calcium-channel blockers, ACE inhibitors, diuretics): the proportion of communities with four BP classes available ranged from 13% in low-income countries to 94% in high-income countries. Similarly, the inability to afford two blood pressure medications affected 31% of households in low-income countries compared to less than 1% in high-income countries (39). The consistent perception of cost as a barrier across countries may be influenced by the socioeconomic status of patient populations served by participants. Participants working in lower income countries were more likely specialist physicians, with 15% family practitioners in LMICs compared to 57% in HICs. These participants may be more likely to serve high income earners in LMICs and hence not be a true representation of the cost barriers to FDC prescription in LMICs.

While FDC may be associated with higher costs, cost-effectiveness analyses of triple (40) and quadruple (41) FDCs indicate that they are cost-effective. A mixed-method study evaluated qualitative data from policy review interviews and quantitative data from the pharmaceutical benefits scheme in Australia. In the study, investigators discovered that FDC therapy led to substantial cost reductions for individual patients, of which, triple FDC yielded the highest median savings of AUD$14.57 (USD$9.52) per prescription (42). The reported cost barrier might reflect participants’ anticipation of patient concerns regarding medication affordability, potentially leading to nonadherence, which disproportionately affects lower-income individuals (43). Participants who prescribed FDC medicines more frequently were less likely to report cost and access as barriers, this suggests that experience with FDC medicines may influence these perceptions. It is possible that some clinicians have perceived costs higher than they are and one systematic review of international studies (24 studies [n = 3321]) indicates that inaccurate prediction of cost is common and more commonly overestimation (44).

Lack of confidence in clinic BP readings, assuming they represent a ‘white coat’ phenomenon, can lead to a ‘wait until next visit’ approach before commencing medication changes (45). This is the likely reason that 40% of clinicians reported they didn’t trust the accuracy of in-clinic BP findings. Clinicians increasingly integrate information from digital innovations with data obtained during clinical encounters (46,47). Additionally, the affordability and accessibility of personal home BP machines has risen in recent years (48). Further exploration of whether this mistrust may be addressed if clinicians had the opportunity to review home BPs was not directly addressed in this study (49).

In general, there was strong agreement with all facilitators, regardless of a countries economic standing. Our results align with evidence demonstrating that providing education to doctors about BP targets and suggestions for up titration is a strategy to overcome treatment inertia (50,51). An RCT that investigated the effect of an outreach coordinator, who met with clinicians to raise awareness of unmet BP goals in comparison with usual care, on treatment inertia found that treatment inertia was reduced 29% in the intervention group in comparison with 11% in the control (51). Social norming dashboards, which display control rates relative to peers, are increasingly being investigated to address treatment inertia (52). This strategy was strongly endorsed by clinicians in our study cohort. Alternative strategies, exemplified by simple treatment protocols like those endorsed by the Global Hearts Initiative may support increased FDC prescribing (53,54). A large proportion of participants agreed that remote monitoring data from devices such as smartwatches would support the prescription of FDC antihypertensive medications. Remote monitoring devices can provide a greater number of BP measurements in different conditions (55). Our results support evidence that increasingly BP measurement will be bolstered with data from personal devices (56,57,58).

Participants reported that FDC combination anti-hypertensive medicines are valuable for patients with a large pill burden or for whom adherence is a problem. These findings corroborate the evidence that adherence to BP lowering medicines is improved with FDCs (59,60,61). Medical adherence and pill burden were the most important factors to consider when thinking about initiating with FDC antihypertensive medicines. While individuals initiated with FDC medicines may show lower persistence (62), coupling FDC medicines with advancements in conversational agents (63), could potentially enhance knowledge and support adherence.

Insights into why there was such variability in responses toward the barriers were sought from the qualitative findings. Our findings mirrored those reported in a systematic review of general practitioners’ (GPs) interviews. In that review, there was inconsistency in the GPs perspectives in relation to prescribing a polypill containing both blood pressure-lowering and cholesterol-lowering drug for CVD prevention (64). For example, one GP advocated for polypills only in high-risk patients whereas another reported that polypill provided an appropriate scalable option for primary prevention of CVD (64). In our results, theme 1, ‘resources dependent,’ mirrored the quantitative results, highlighting cost and access as limiting factors contributing to whether these medicines are prescribed. Theme 2, ‘challenges of a fixed dose,’ suggests that a loss of autonomy when using FDC medicines is a barrier to their prescription. While we did not specifically ask about autonomy, this aligns with previous research highlighting the negative impact on prescribing when health professionals lack the flexibility to titrate the dose (64). Theme 3, ‘insufficient information,’ was interpreted as a key factor contributing to the inconsistency in responses to the barriers. A qualitative study that investigated the attitudes of primary healthcare professionals to a polypill containing both blood pressure-lowering and cholesterol-lowering medicine found health professionals needed evidence demonstrating the polypill to be safe, effective and beneficial before prescribing a polypill (65).

### Strengths and limitations

The current study provides information from an international cohort of practicing clinicians on the perceived barriers and enablers to FDCs use for hypertension and also the perceived value of FDCs. However the study has some limitations, the key one being that it is difficult to obtain a representative sample of international clinicians, and while the survey was provided in multiple languages and the snowball recruitment applied, there may have been bias clinicians with more positive perceptions of FDCs. Survey piloting was conducted among colleagues within our professional network in Australia, potentially limiting identification of survey limitations that may arise in a broader population. Second, the survey instrument was not validated against real-world prescribing data, limiting the assessment of self-reported practices. Third, we included doctors only, we did not recruit nurses and other health professionals as we were not confident that we would recruit sufficient number to draw meaningful comparisons. Finally, data aggregation into higher and lower-income groups obscured potential differences in perceived cost and access barriers, for example, we noted differences between Australia (high-income) and Malaysia (upper-middle-income). A larger, multinational sample is required to validate these findings.

## Conclusions

International clinicians see value in FDCs particularly to enhance adherence and manage pill burden. Yet cost was a strong and consistent obstacle to prescribing FDC antihypertensive medications. Both clinician nudges and enhanced education peer support initiatives were both supported as having the potential to foster improved FDC use.

## Data Accessibility Statement

De identified data is available from the corresponding author on request.

## Additional File

The additional file for this article can be found as follows:

10.5334/gh.1353.s1 Supplementary Material. Supplementary Material 1 to 5.
