# Supplementary material for: A Cross-Sectional Survey of Fixed-Dose Combination Antihypertensive Medicine Prescribing in Twenty-Four Countries, Including Qualitative Insights
Source: Glob Heart. 2024 Sep 12;19(1):73. doi: 10.5334/gh.1353 (PMC11396169; doi:10.5334/gh.1353)
Supplement: Supplementary Material. — Supplementary Material 1 to 5. [file gh-19-1-1353-s1.pdf]

A multi-methods cross-sectional survey of Fixed dose combination therapy antihypertensive medicine prescribing in twenty-four countries.

Supplementary Material

1. STROBE checklist……………………………...……………………………………………..…………2
2. Survey questions………………………………………………………………………………………4
3. Table 1- List of participating countries…………………………………...…………………..……….9
4. Figure 1-Level of agreement with each barrier and facilitator for doctors in high and upper middle income or lower-middle and low income countries …..…………………..……………………………10
5. Table 2-Beliefs about prescribing appropriateness…………………….…………………………….11

1.STROBE checklist

STROBE Statement—Checklist of items that should be included in reports of ***cross-sectional studies***

	Item No	Recommendation	Page No
**Title and abstract**	1	(*a*) Indicate the study's design with a commonly used term in the title or the abstract	1
		(*b*) Provide in the abstract an informative and balanced summary of what was done and what was found	2
**Introduction**			
Background/rationale	2	Explain the scientific background and rationale for the investigation being reported	3
Objectives	3	State specific objectives, including any prespecified hypotheses	3
**Methods**			
Study design	4	Present key elements of study design early in the paper	4
Setting	5	Describe the setting, locations, and relevant dates, including periods of recruitment, exposure, follow-up, and data collection	4
Participants	6	(*a*) Give the eligibility criteria, and the sources and methods of selection of participants	4
Variables	7	Clearly define all outcomes, exposures, predictors, potential confounders, and effect modifiers. Give diagnostic criteria, if applicable	5
Data sources/ measurement	8*	For each variable of interest, give sources of data and details of methods of assessment (measurement). Describe comparability of assessment methods if there is more than one group	*5*
Bias	9	Describe any efforts to address potential sources of bias	N/A
Study size	10	Explain how the study size was arrived at	N/A
Quantitative variables	11	Explain how quantitative variables were handled in the analyses. If applicable, describe which groupings were chosen and why	5
Statistical methods	12	(*a*) Describe all statistical methods, including those used to control for confounding	5
		(*b*) Describe any methods used to examine subgroups and interactions	5
		(*c*) Explain how missing data were addressed	N/A
		(*d*) If applicable, describe analytical methods taking account of sampling strategy	N/A
		(*e*) Describe any sensitivity analyses	N/A
**Results**			
Participants	13*	(a) Report numbers of individuals at each stage of study—eg numbers potentially eligible, examined for eligibility, confirmed eligible, included in the study, completing follow-up, and analysed	6
		(b) Give reasons for non-participation at each stage	7
		(c) Consider use of a flow diagram	7
Descriptive data	14*	(a) Give characteristics of study participants (eg demographic, clinical, social) and information on exposures and potential confounders	8
		(b) Indicate number of participants with missing data for each variable of interest	7
Outcome data	15*	Report numbers of outcome events or summary measures	8

Main results	16	(*a*) Give unadjusted estimates and, if applicable, confounder-adjusted estimates and their precision (eg, 95% confidence interval). Make clear which confounders were adjusted for and why they were included	8
		(*b*) Report category boundaries when continuous variables were categorized	8
		(*c*) If relevant, consider translating estimates of relative risk into absolute risk for a meaningful time period	N/A
Other analyses	17	Report other analyses done—eg analyses of subgroups and interactions, and sensitivity analyses	9
**Discussion**			
Key results	18	Summarise key results with reference to study objectives	11
Limitations	19	Discuss limitations of the study, taking into account sources of potential bias or imprecision. Discuss both direction and magnitude of any potential bias	12
Interpretation	20	Give a cautious overall interpretation of results considering objectives, limitations, multiplicity of analyses, results from similar studies, and other relevant evidence	11
Generalisability	21	Discuss the generalisability (external validity) of the study results	12
**Other information**			
Funding	22	Give the source of funding and the role of the funders for the present study and, if applicable, for the original study on which the present article is based	6

*Give information separately for exposed and unexposed groups.

Note: An Explanation and Elaboration article discusses each checklist item and gives methodological background and published examples of transparent reporting. The STROBE checklist is best used in conjunction with this article (freely available on the Web sites of PLoS Medicine at http://www.plosmedicine.org/, Annals of Internal Medicine at http://www.annals.org/, and Epidemiology at http://www.epidem.com/). Information on the STROBE Initiative is available at www.strobe-statement.org.

2. Survey Questions

Eligibility

1. Are you a medical doctor?
   a. Yes
   b. No

2. As part of your usual medical practice do you prescribe antihypertensive medications to patients with high blood pressure?
   a. Yes
   b. No

(Participants who answer No to either question are not eligible and directed to the end of the survey.)

Barriers and Facilitators

We will ask you to rate your level of agreement or disagreement with statements about barriers and facilitators to prescribing fixed-dose combination antihypertensive medications.

Barriers - System and clinician level factors

1. Appointment time: *You have insufficient time during consultations to explain medication changes to the patient.*
   a. Strongly disagree
   b. Disagree
   c. Neutral
   d. Agree
   e. Strongly agree

2. Access: *Your patients do not have access to fixed-dose combination antihypertensive medications where you work.*
   a. Strongly disagree
   b. Disagree
   c. Neutral
   d. Agree
   e. Strongly agree

3. Lack of confidence in BP measurement: *Blood pressure measurements taken in the clinic setting are inaccurate.*
   a. Strongly disagree
   b. Disagree
   c. Neutral
   d. Agree
   e. Strongly agree

4. Cost to the medical practice or hospital that you work in: *Fixed-dose combination antihypertensive medications are too expensive for your medical practice to support.*
    a. Strongly disagree
    b. Disagree
    c. Neutral
    d. Agree
    e. Strongly agree
    f. Not applicable

<u>Patient level factors</u>

5. Side effects: *Your patients are concerned about experiencing more side effects (for example dizziness, headache) when taking fixed-dose combination antihypertensive medications compared to adding medicines sequentially.*
    a. Strongly disagree
    b. Disagree
    c. Neutral
    d. Agree
    e. Strongly agree

6. Adherence: *You are concerned that your patients are less likely to adhere to a fixed-dose combination medication regimen than to multiple separate prescriptions.*
    a. Strongly disagree
    b. Disagree
    c. Neutral
    d. Agree
    e. Strongly agree

<u>Facilitators – System and clinician level factors</u>

1. Clinician nudge. *A clinician nudge that provides a prompt in the electronic health record during the patient visit to prescribe fixed-dose combination antihypertensive medications would support the prescription of those medications.*
    a. Strongly disagree
    b. Disagree
    c. Neutral
    d. Agree
    e. Strongly agree

2. Education or guideline updates. *Providing education and feedback on prescribing patterns compared with peers focusing on fixed-dose combination antihypertensive medications would support the prescription of those medications.*
    a. Strongly disagree
    b. Disagree

c. Neutral
d. Agree
e. Strongly agree

3. Additional BP measurement data. *Having access to blood pressure data from remote monitoring devices such as smartwatches would support the prescription of fixed-dose combination antihypertensive medications.*

f. Strongly disagree
g. Disagree
h. Neutral
i. Agree
j. Strongly agree

<u>Patient level factors</u>

4. Improve health literacy. *Patient information such as leaflets and flyers in clinics focusing on fixed-dose combination antihypertensive medications would support prescription of those medications.*

a. Strongly disagree
b. Disagree
c. Neutral
d. Agree
e. Strongly agree

Demographics

1. What is your age?
    a. Under 25 years
    b. 26-35 years
    c. 36-45 years
    d. 46-55 years
    e. 56-65 years
    f. Over 66 years
2. What is your gender?
    a. Male
    b. Female
    c. Non-binary/third gender
    d. Prefer not to say
3. How many years have you been practicing medicine?
    (Enter number to the nearest year)
4. In which country did you do your medical training? (Drop down list of countries)
5. In which country do you work? (Drop down list of countries)

Experience

1. What is your specialty?

- Cardiologist
- Endocrinologist
- Family Practitioner/General Practitioner
- Geriatrician
- Nephrologist

Other (Please specify)

2. How many patients do you treat (initiate or adjust) with antihypertensive medicines, per week?

    - 0-20
    - 21-40
    - 41-60
    - 61-80
    - 81-100
    - 100+
    -

The next set of questions ask you about your current prescribing practices.

Considering your last working week, what percentage of the patients you saw are on the following antihypertensive regimens:
a) Single drug monotherapy
b) Single pill combination of 2 medicines
c) Single pill combination of 3 or more medicines
d) Two blood pressure lowering medicines separately
e) Three or more blood pressure lowering medicines separately

Thinking about the patients you saw last week on single drug monotherapy, what percentage are on each of the following;
a) ACE inhibitor or ARB (eg. ramipril or telmisartan)
b Dihydropyridine calcium channel blocker (eg. amlodipine)
c) Thiazide-like diuretic (eg hydrochlorothiazide)
e) Loop diuretic (eg furosemide)
d) Beta blocker (eg atenolol, metoprolol)
e) Other blood pressure lowering medicine

Caseload

Thinking about the last 10 patients you saw about hypertension (which may or may not be well controlled), how many did you prescribe a fixed-dose combination antihypertensive medication?
Enter number on scale 0-10

Value and importance

In your opinion in what situations do fixed-dose combination antihypertensive medicines have value?

1= very low value, 2= low value, 3=moderate value, 4= high value, 5=very high value

- Patients with a large pill burden
- Patients at high cardiovascular risk
- Patients who are non-adherent or in whom adherence is an issue
- Patients with BP >160/ 100 on maximal dose of a single agent
- Patients with BP >160/100 on no medication
- Patients with very high CV risk with BP >130/90

How important do you consider each of these factors in your decision to initiate fixed-dose combination therapy with your patients?

1= not at all important, 2= slightly important, 3=moderately important, 4= very important, 5= extremely important.

- The patient’s BP is not at target
- Pill burden
- Overall risk for cardiovascular disease
- Frailty and risk of falls
- Cost of treatment
- Medical adherence

Additional comments

Optional question

What additional comments do you have regarding barriers and facilitators to the use of fixed-dose combination antihypertensive medications?

3.Table 1-List of participating countries

**Country where participant trained**		**Country where participant works**	
**Country**	**Number of responses**	**Country**	**Number of responses**
Argentina	1	Argentina	1
Armenia	1	Australia	21
Australia	21	Bahamas	1
Bahrain	1	Bahrain	2
Benin	1	Belgium	1
CÃ´te d'Ivoire	2	CÃ´te d'Ivoire	2
Cameroon	2	Cameroon	5
Canada	2	Honduras	1
China	1	India	34
Cuba	1	Jamaica	1
Egypt	1	Malaysia	64
Honduras	1	Mali	8
Iceland	1	Mexico	3
India	38	Mozambique	1
Ireland	4	New Zealand	6
Italy	1	Peru	5
Jamaica	1	Saint Kitts	1
Malaysia	50	Saudi Arabia	4
Mali	5	Singapore	2
Mexico	2	Spain	1
Morocco	4	Sudan	2
Mozambique	1	United Kingdom of Great Britain and Northern Ireland	3
New Zealand	3	USA	5
Peru	5	Viet Nam	5
Russia	2		
Saudi Arabia	1		
Singapore	1		
Spain	1		
Sudan	2		
United Kingdom of Great Britain and Northern Ireland	10		
USA	7		
Viet Nam	5		

4.Figure 1- Level of agreement with each barrier and facilitator for doctors in high and upper middle income or lower-middle and low income countries

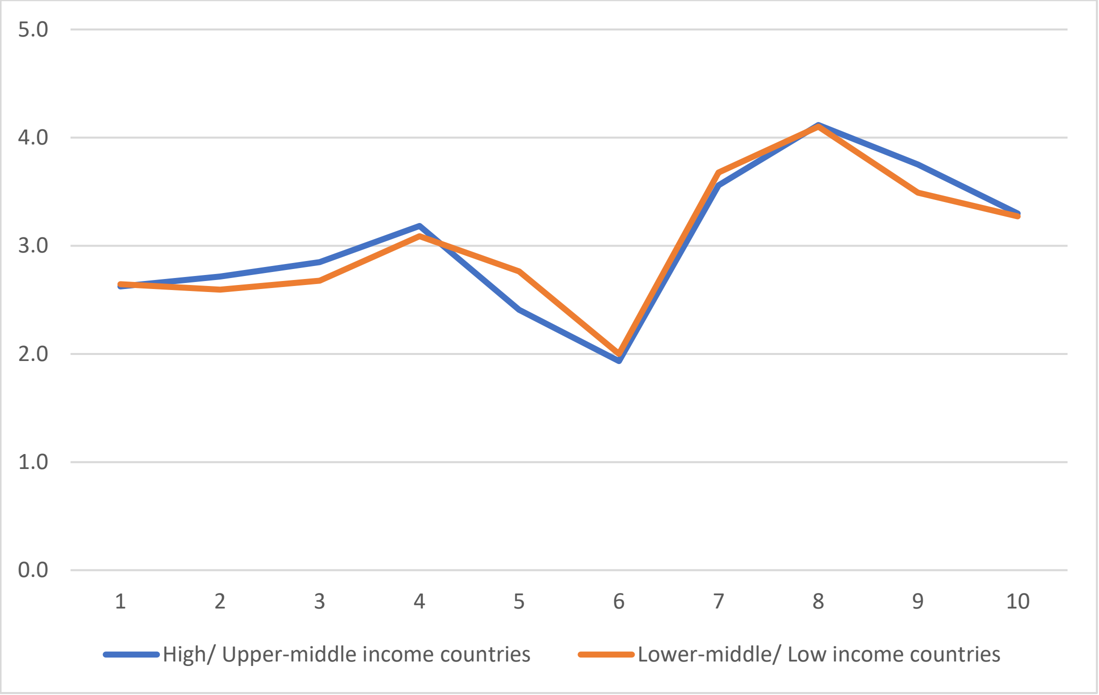

5. Table 2- Beliefs about prescribing appropriateness

In what situations does a fixed-dose combination antihypertensive medication have value?*	All (including data where country of work was not available)	High and upper middle income	Lower-middle and low income·
Patients with a large pill burden [n; mean (SD)]	169; 4·2 (0·9)	117; 4.3 (0·9)	52; 4·0 (0·9)
Patients with a high cardiovascular risk [n; mean (SD)]	169; 3·7 (0·9)	117; 3·7(0·9)	52; 3·7 (0·9)
Patients who are non-adherent or in whom adherence is an issue [n; mean (SD)]	169; 4·2 (0·8)	117; 4·3 (0·9)	52; 4·1 (0·7)
Patients with blood pressure >160/100 on maximal dose of a single agent [n; mean (SD)]	169; 3·9 (1·0)	117; 3·9 (1·0)	52; 4·0 (0·9)
Patients with blood pressure >160/100 on no medication [n; mean (SD)]	169; 3·3 (1·2)	117; 3·4 (1·2)	52; 3·2 (1·1)
Patients with very high cardiovascular risk with blood pressure >130/90 [n; mean (SD)]	169; 3·3 (1·0)	117; 3·3 (1·0)	52; 3·4 (1·0)
How important do you consider each of these factors in your decision to initiate fixed-dose combination antihypertensive therapy with your patients?**			
The patient's blood pressure is not at target [n; mean (SD)]	169; 3·7 (1·0)	117; 3·7 (0·9)	52; 3·7 (1·0)
Pill burden [n; mean (SD)]	169; 4·2 (0·8)	117; 4·3 (0·8)	52; 4·0 (0·8)
Overall risk for cardiovascular disease [n; mean (SD)]	169; 3·7 (1·0)	117; 3·7 (1·0)	52; 3·6 (0·9)
Frailty and risk of falls [n; mean (SD)]	169; 3·4 (1·1)	117; 3·5 (1·1)	52; 3·3 (1·1)
Cost of treatment [n; mean (SD)]	169; 3·8 (1·1)	117; 3·8 (1·2)	52; 3·8 (1·0)
Medical adherence [n; mean (SD)]	169; 4·2 (0·8)	117; 4·2 (0·9)	52; 4·3 (0·7)

*Rated on 1-5 scale with a range from 1= Very low value to 5= Very high value
** Rated on 1-5 scale with a range from 1= Not at all important to 5= Extremely important
